# Integrating the Local Property and Topological Structure in the Minimum Spanning Tree Brain Functional Network for Classification of Early Mild Cognitive Impairment

**DOI:** 10.3389/fnins.2018.00701

**Published:** 2018-10-08

**Authors:** Xiaohong Cui, Jie Xiang, Bin Wang, Jihai Xiao, Yan Niu, Junjie Chen

**Affiliations:** ^1^College of Information and Computer, Taiyuan University of Technology, Taiyuan, China; ^2^Center of Information Management and Development, Taiyuan University of Technology, Taiyuan, China

**Keywords:** minimum spanning tree, local property, topological structure, the multikernel SVM, early mild cognitive impairment, classification

## Abstract

Abnormalities in the brain connectivity in patients with neurodegenerative diseases, such as early mild cognitive impairment (EMCI), have been widely reported. Current research shows that the combination of multiple features of the threshold connectivity network can improve the classification accuracy of diseases. However, in the construction of the threshold connectivity network, the selection of the threshold is very important, and an unreasonable setting can seriously affect the final classification results. Recent neuroscience research suggests that the minimum spanning tree (MST) brain functional network is helpful, as it avoids the methodological biases while comparing networks. In this paper, by employing the multikernel method, we propose a framework to integrate the multiple properties of the MST brain functional network for improving the classification performance. Initially, the Kruskal algorithm was used to construct an unbiased MST brain functional network. Subsequently, the vector kernel and graph kernel were used to quantify the two different complementary properties of the network, such as the local connectivity property and the topological property. Finally, the multikernel support vector machine (SVM) was adopted to combine the two different kernels for EMCI classification. We tested the performance of our proposed method for Alzheimer's Disease Neuroimaging Initiative (ANDI) datasets. The results showed that our method achieved a significant performance improvement, with the classification accuracy of 85%. The abnormal brain regions included the right hippocampus, left parahippocampal gyrus, left posterior cingulate gyrus, middle temporal gyrus, and other regions that are known to be important in the EMCI. Our results suggested that, combining the multiple features of the MST brain functional connectivity offered a better classification performance in the EMCI.

## Introduction

Alzheimer's disease (AD) is a common progressive neurodegenerative disease that affects the nervous system. In 2018, the number of AD patients in the United States will reach 5.7 million and the cost of treatments will reach 277 billion, causing great economic losses to the families and the society (Alzheimer's Association, 2018). Therefore, in the early stage, such as early mild cognitive impairment (EMCI), it is important to find the symptoms of the disease and develop strategies to treat it. However, the subtle differences in the cognitive function between the EMCI and normal control (NC) make it difficult to diagnose the EMCI. Therefore, it is very important to propose a framework to identify the individuals with EMCI from NC.

At present, the brain functional magnetic resonance imaging (fMRI) data is represented as a brain network composed of nodes and edges (Lópezsanz et al., 2017). Through the analysis and study of the brain network, the brain functional network of the mild cognitive impairment (MCI) patients exhibit abnormal local properties and topological structures (Supekar et al., 2008; Sanz-Arigita et al., 2010; Petrella et al., 2011; Liu et al., 2012; Wang et al., 2017; Yan et al., 2018). Jie et al. (2014b) constructed an undirected functional brain network of NC and MCI, and extracted the topological features to classify the two groups of subjects, where abnormal regions were found in the brain network including those in the hippocampus, amygdala, and the inferior temporal gyrus. Khazaee et al. (2016) also constructed an undirected brain network of NC, MCI, and AD groups by using 264 putative functional areas. Network topology attributes were extracted as classification features to be used in the classification of three groups of subjects. The result showed that this method was able to accurately classify three groups (i.e., NC, MCI, and AD) with an accuracy of 88.4%, and it was found that the left posterior central gyrus, the right inferior temporal gyrus, the left lingual gyrus, the right middle frontal gyrus, and the right thalamus were significantly different from the normal elderly. Wee et al. (2016) designed a disease identification framework based on the estimated temporal networks, and analyzed the group differences in the level network property. Yu et al. (2016) studied the directed functional connectivity using the Granger causality analysis (GCA), and found that the posterior cingulate cortex (PCC) in the Default Mode Network (DMN) showed directional disorders in receiving and transmitting information.

A common problem in the above studies was the use of a single type of network property for the MCI, and NC classification, such as the local connectivity or global topological properties. In order to improve the accuracy in the MCI diagnosis, Jie et al. (2014a) extracted local connectivity and global topological properties from five different threshold brain networks and combined these properties in the classification of MCI and NC. However, this may affect the final classification performance to some extent, since we need to set a threshold for the original weighted network in the construction of the threshold brain network. In 2015, Tewarie et al. proposed the minimum spanning tree (MST) as an unbiased approach in the construction and the analysis of the brain networks (Tewarie et al., 2015). MST method preserves the core framework of the networks while voiding the influences of the threshold. It does not only reduce the computational cost, but also guarantees the network's neurological interpretability. In 2006, Lee et al. applied the MST to brain network for the first time, and MST was widely applied in the research and development of many kinds of neuropsychiatric disorders (Lee et al., 2006; Boersma et al., 2012; Demuru et al., 2013; Stam et al., 2014).

**Figure 1:**
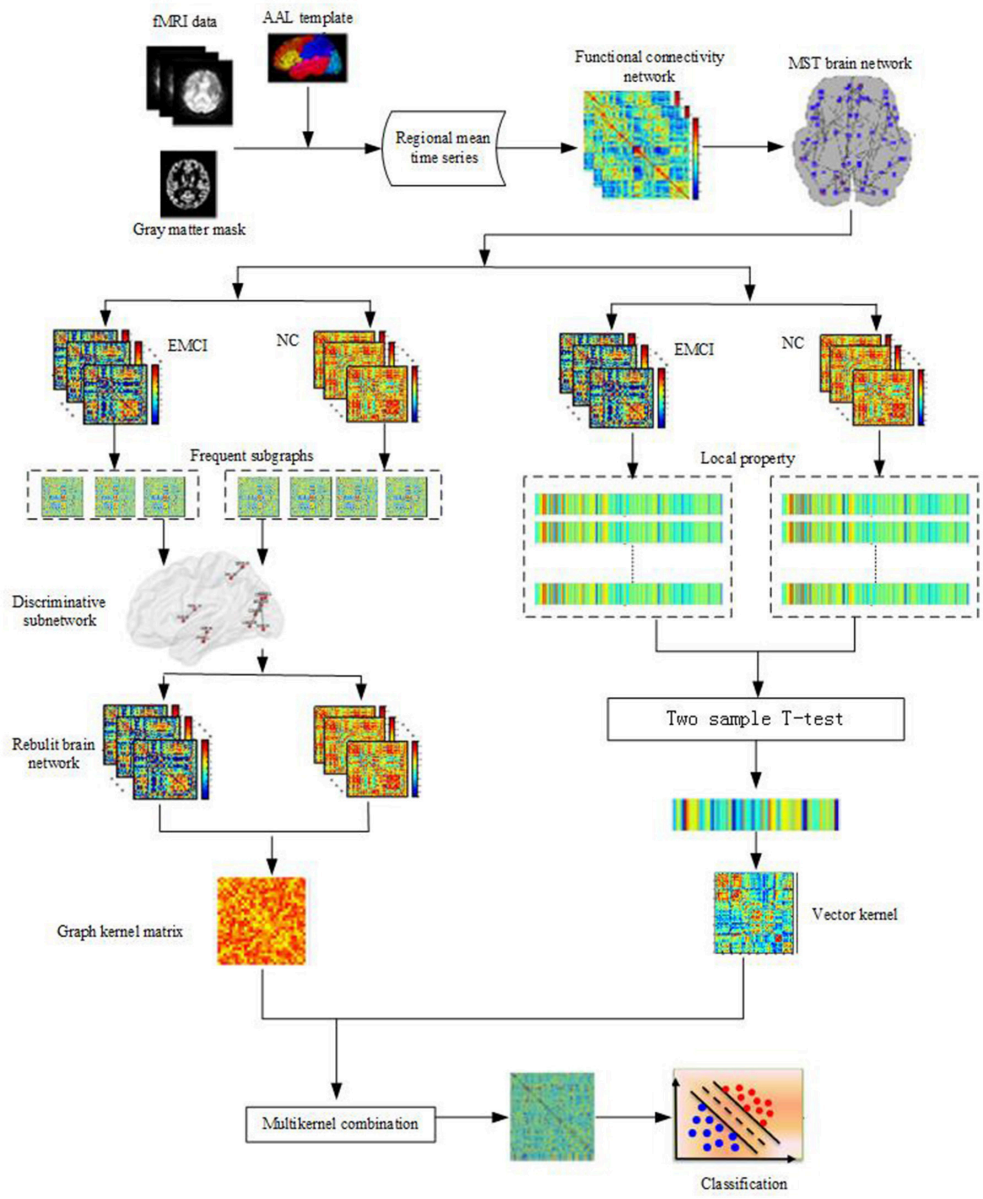
Framework of proposed method. Firstly, data preprocessing of fMRI; Then the MST brain functional network is constructed, the local property and topological structure property of the MST brain functional network are extracted, Finally, the two features are combined to classify by using Multi-kernel SVM.

Accordingly, in this paper, based on an unbiased MST brain network, a classification framework combining the local properties and topological structures is proposed. Figure 1 illustrates the framework of our proposed method. Initially, the MST brain functional network was constructed, then the local property and topological structure property of the MST brain functional network were extracted, and the two features were combined to identify the EMCI from the NC. Experiments showed that the classification framework not only realized the complementation of local and topological structure properties, but also improved the classification performance.

## Materials and methods

### Data acquisition and preprocessing

The data used in this study was from the Alzheimer's Disease Neuroimaging Initiative (ADNI) database at website http://adni.loni.usc.edu/. A total of 60 subjects were selected from ADNI-2 database, including 32 EMCI patients and 28 NC. Table 1 shows the demographics of all participants. A 3.0 T scanner (Philips Medical Systems) was used to acquire resting-state BOLD fMRI scans of all subjects. The scanning parameters were set as follows: repetition time (TR) = 3,000 ms; echo time (TE) = 30 ms; slice thickness = 3.3 mm; flip angle = 80°; slice number = 48 and 140 time points. During scanning, all the subjects were instructed to keep their eyes closed.

**Table 1:** The demographics of all participants.

**Group**	**EMCI**	**NC**
No. of subjects (M/F)	17/15	11/17
Age (mean ± SD)	72.1 ± 6.0	74.3 ± 6.2
MMSE (mean ± SD)	27.7 ± 1.9	28.9 ± 1.3
CDR (mean ± SD)	0.48 ± 0.1	0 ± 0

*EMCI, Early Mild Cognitive Impairment; NC, Normal Control; MMSE, Mini-Mental State Examination; CDR, Clinical Dementia Rating; M, Male; F, Female*.

Many preprocessing steps of the fMRI images were performed using Data Processing Assistant for Resting-State fMRI (DPARSF; Yan and Zang, 2010), Statistical Parametric Mapping (SPM12; http://www.fil.ion.ucl.ac.uk/spm), and the Resting-State fMRI Data Analysis Toolkit (REST 1.8) packages (Song et al., 2011). Specifically, the first 10 time points of each subject were removed; slice-timing correction and image realignment were carried out on the remaining 130-time points. Because the brain size, shape, orientation, and gyral anatomy of each subject is different, the fMRI data of each subject was usually normalized into the Montreal Neurological Institute (MNI) space (resampled into 3 × 3 × 3 mm^3^ voxels) by using a unified segmentation on the T1 image. Then, the linear trends of the time courses were removed, and the effect of nuisance covariates was removed by signal regression using the global signal, the six motion parameters, the cerebrospinal fluid (CSF) and white matter (WM) signals. Temporal filtering (0.01 Hz < f < 0.08 Hz) was applied. Lastly, since we used only gray matter (GM) tissue to construct the functional connectivity network, the gray matter mask was used to mask the corresponding fMRI images to eliminate the possible effects from CSF and WM.

### Methods

The key technologies in this paper included: Kruskal algorithm (Kruskal, 1956), graph-based Substructure pattern mining (gSpan; Yan and Han, 2002), the local feature selection, the discriminative subgraph algorithm selection and the multikernel learning technique (Zhang D. et al., 2011). Firstly, the unbiased brain functional network was constructed using the Kruskal algorithm, and betweenness was extracted as the local property. Then, frequent subgraphs were mined from the brain network using the gSpan algorithm, and the discriminative subgraphs of brain networks were extracted as the topological property. Lastly the local property and topological property were combined to classify the EMCI.

#### Construction of the unbiased MST brain functional network

The brain network can be abstracted into a graph. The construction of the brain network involved the determination of the nodes and edges in the graph. In this study, construction steps of MST brain functional network included:

1. Definition of node: We parcellated the gray-matter masked voxels into 90 regions of interest (ROIs) by the Automated Anatomical Labeling (AAL) template (Tzourio-Mazoyer et al., 2002). A ROI is a node of the brain network. Therefore, the brain network consisted of 90 nodes.
2. Definition of edge: The average value of the fMRI time series of all voxels in each ROI is considered as the average time series of the node, and the Pearson correlation coefficient between the pair of nodes is taken as the weight of the connected edges. So, a functional full connected network is constructed for each subject. Moreover, in order to extract the meaningful network measures, we removed all negative correlations from the obtained connectivity networks.
3. Construction of unbiased brain functional network: In order to construct an unbiased brain network, we used the Kruskal algorithm to construct the MST brain network. MST is a weighted graph that connects the nodes together, without any cycles, and with the minimum weight. Since we were only interested in the strongest connections in the brain network, the Kruskal algorithm was used to construct a weighted graph that connects all the nodes together, without any cycles and with the maximum weight. The algorithm sorted initially, all the correlation coefficients into descending order, and then connected the edges with the largest correlation coefficients that were added successively until all nodes were connected in an acyclic subnetwork. In this process, if the addition of a link formed a loop, this link was ignored.

#### Local property of the MST brain functional network

##### Local property

Betweenness is an important local property in the MST, and it was also recognized as the most relevant feature in the classification between the MCI and the NC (Ebadi et al., 2017). So betweenness was extracted as a feature. Betweenness of node was defined as the number of all the shortest paths through this node.

The betweenness *bc*_*i*_ of the node *i* was defined as (Tewarie et al., 2015):

(1) $$\begin{array}{l} bc_{i}=\frac{1}{\left(n-1\right)\left(n-2\right)}\underset{\begin{array}{c} h,j\in V\\ h\neq j,h\neq i \end{array}}{\sum }\frac{\rho_{hj}^{i}}{\rho_{hj}} \end{array}$$

Where ρ_*hj*_ represents the number of the shortest paths between the node *h* and *j*; $\rho_{hj}^{i}$ represents the number of the shortest paths between the node *h* and *j* through the node *i*; V represents the set of nodes; and n represents the number of nodes.

##### Discriminative brain regions selection

We calculated the betweenness of each node in the MST functional network. To select the most discriminative brain region, two sample *t*-test was used. The brain regions with *p* < 0.05 were selected as the discriminative brain regions.

##### Linear kernel

The betweenness of the discriminative brain regions composed a feature vector representing the local property of a brain network. We measured the similarity of two functional connectivity networks in term of local property by using linear kernel as follows:

(2) $$\begin{array}{l} {\text{k}}_{v}\left(\text{x},\text{y}\right)={\text{x}}^{\text{T}}\text{y} \end{array}$$

Where x and y represent the feature vectors from two subjects, respectively.

#### Topological property of brain network

##### Frequent subgraph mining

In order to capture the differences in the topological structure of the brain networks, this study uses the gSpan algorithm to extract the frequent subgraphs from the brain network, and the most discriminative subgraphs were selected.

Definition 1 (Undirected labeled network): For an undirected labeled network G = (V, E, L), V represents the set of nodes; *E*⊆*V*×*V*, the set of edges; L, the set of labels.

Definition 2 (Subnetwork): Given two undirected labeled networks G = (V, E, L) and *G*_*s*_ = (*V*_*s*_, *E*_*s*_, *L*_*s*_), if *V*_*s*_ ⊆ *V, L*_*s*_ ⊆ *L and E*_*s*_ ⊆ *E*, G_s_ is a subnetwork of G.

Definition 3 (Subnetwork frequency): For a given network set 𝔾, 𝔾 = {*G*_1_, *G*_2_, ⋯*G*_*n*_}, n is the number of networks. The frequency f_q_ of a subnetwork g_s_ is defined in Equation (3):

(3) $$\begin{array}{l} {\text{f}}_{\text{q}}\left({\text{g}}_{\text{s}}|𝔾\right)=\frac{\left|{\text{g}}_{\text{s}}\text{ is subgraph of G and G }\in 𝔾\right|}{\left|𝔾\right|} \end{array}$$

where |𝔾| presents the number of networks.

Definition 4 (Frequent subnetwork mining): For a given undirected labeled network set 𝔾 and frequency thresholding value s where 0 ≤ s ≤ 1, the process of finding all subnetworks of 𝔾 with the frequency of at least s is called frequent subnetwork mining.

##### Discriminative subgraphs selection

In fact, there exist a large number of frequent subgraphs in a network, but only a small portion of the frequent subgraphs have the discriminability. Therefore, the most discriminative subgraphs were selected by using the further feature selection method based on their respective frequency difference (Wang et al., 2015).

The frequency difference *D*(*g*_*s*_) of subgraph *g*_*s*_ is defined in Equation (4):

(4) $$\begin{array}{l} \text{D}\left({\text{g}}_{\text{s}}\right)=|{\text{f}}_{\text{q}}\left({\text{g}}_{\text{s}}\text{|}{\text{G}}_{\text{p}}\right)-{\text{f}}_{\text{q}}\left({\text{g}}_{\text{s}}\text{|}{\text{G}}_{\text{n}}\right)| \end{array}$$

Where *G*_*p*_ denotes the set of frequent subgraphs for positive samples, and *G*_*n*_ denotes the set of frequent subgraphs for negative samples.

The greater the frequency difference, the stronger is the discriminability. The frequency difference of frequent subgraphs were calculated and then the frequency difference threshold T was set. The subgraphs with a frequency differences greater than T were considered to be the most discriminative subgraphs.

Then, the brain network was reconstructed using the most discriminative subgraphs. Specifically, for a network, we only needed to delete the edges that did not appear in any discriminative subgraphs. In this way, the topology of the brain network and the discriminative subgraphs was preserved.

##### Graph kernel

The brain network is a complex structural dataset. The traditional feature extraction methods cannot deal with the complex topological features of the brain network. Graph kernel can map data from the original graph space to the feature space, and the similarity between the two graphs is further measured by comparing the topological structure of the graph. Therefore, the graph kernel establishes a bridge between the graph data and many kernel-based learning algorithms, and has been successfully applied in the fields of computer vision (Camps-Valls et al., 2010) and bioinformatics (Zhang Y. et al., 2011).

Recent research has shown that the Weisfeiler-Lehman (WL) subtree kernel (Shervashidze et al., 2011) could be efficiently computed in time O(|E|), and was a suitable option for brain graph classification (Vega-Pons et al., 2014). In this paper, we have used the WL subtree-based kernel method to measure the topological similarity between the brain networks. For a pair of brain networks G and H, the basic processes of WL subtree-based kernels were as follows:

1. Initially, every vertex of a graph was labeled with a degree of that node.
2. At each iteration, the label of each node was augmented in the graph by a sorted set of node labels of neighboring nodes, and these augmented labels were compressed into a new short label.
3. This process proceeded iteratively until the node label sets of two graphs differed, or the number of iteration reached the maximum h.
4. The WL subtree-based kernel on two graphs G and H is defined in Equation (5):

(5) $$\begin{array}{l} {\text{k}}_{g}(\text{G},\text{H})=<\varphi \left(\text{G}\right),\varphi \left(\text{H}\right)> \end{array}$$

Where

$$\begin{array}{l} \varphi \left(\text{G}\right)=(\sigma_{0}\left(\text{G},{\text{s}}_{01}\right),\cdots ,\sigma_{0}\left(\text{G},{\text{s}}_{0\left|{\text{L}}_{0}\right|}\right),\cdots ,\\ \;\sigma_{\text{h}}\left(\text{G},{\text{s}}_{\text{h}1}\right),\cdots ,\sigma_{\text{h}}\left(\text{G},{\text{s}}_{\text{h}\left|{\text{L}}_{\text{h}}\right|}\right)) \end{array}$$

$$\begin{array}{l} \varphi \left(\text{H}\right)=(\sigma_{0}\left(\text{H},{\text{s}}_{01}\right),\cdots ,\sigma_{0}\left(\text{H},{\text{s}}_{0\left|{\text{L}}_{0}\right|}\right),\cdots ,\\ \;\sigma_{\text{h}}\left(\text{H},{\text{s}}_{\text{h}1}\right),\cdots ,\sigma_{\text{h}}\left(\text{H},{\text{s}}_{\text{h}\left|{\text{L}}_{\text{h}}\right|}\right)) \end{array}$$

σ_*i*_(*G, s*_*i,j*_) and σ_*i*_(*H, s*_*i,j*_) is the numbers of occurrences of the label *s*_*i,j*_ in G and H, respectively, *s*_*i,j*_ denotes the label of *i*-th node in iteration *j*. |*L*_*i*_ | is the number of labels in the iteration *i*, *L*_*i*_ denotes the label set of G, and H in iteration *i*, *L*_0_ represents the initial labels set of G and H. K is the kernel matrix of *n*×*n*, n is the number of brain networks.

#### The multikernel SVM

Recent studies on multikernel SVM has proved that the multikernel integration not only improves the accuracy of classification, but also improves the interpretability of the results (Lanckriet et al., 2002). Neuroimaging studies have also shown that multikernel integration can systematically aggregate different kernels into a single mode (Wee et al., 2012).

In this paper, we consider two types of kernels, i.e., the linear kernel and the graph kernel. We assumed that these kernels could provide the complementary information for EMCI identification.

Firstly, as this research uses two different types of kernel, normalization was done individually. Then we used a multi-kernel SVM technique to linearly combine the two kernels, as shown in Equation (6):

(6) $$\begin{array}{l} \text{K}\left(G,\text{H}\right)=\beta {\text{k}}_{v}\left(\text{x},\text{y}\right)+(1-\beta ){\text{k}}_{g}(\text{G},\text{H}) \end{array}$$

Where G and H are two MST functional networks, *k*_*g*_(*G, H*) is a graph kernel of G and H, x and y are their local feature vectors of G and H, *k*_*v*_(*x, y*) is a linear kernel, and β is a nonnegative weighting parameter.

Once β was determined, we used the traditional single-kernel SVM (Chang and Lin, 2011) for the classification.

#### Methodology

On the basis of pre-processing, Kruskal algorithm was used to construct an unbiased brain network. The betweenness of the node was extracted and the feature was selected from the training set by using two sample *t*-test. In addition, the linear kernel was adopted as the vector kernel. Then using gSpan algorithm (s is set to 0.7), the frequency subgraphs of brain network was mined and the most discriminative subgraphs (the frequency differences >0.13) were selected. Subsequently, we used the WL subtree kernel (h and n are set as 2 and 1, respectively) to extract the topological features of the reconstructed brain network, and, the optimal weighting parameter β was obtained from the training set via a grid search (the range from 0 to 1 at a step size of 0.1). Finally, the conventional SVM framework was used to identify the EMCI from NCs. All experiments are performed using 10-fold cross-validation. Specially, the subject dataset was randomly divided into 10 parts, one of which was left as the testing set, while the remaining nine were used as training sets. The feature selection was carried out on the training set, and the selected discriminative features were used to build the classification model, then this model was used to classify on the testing set. Ten-fold cross-validation was preformed 50 times. Finally, we computed the arithmetic mean of the 50 repetitions as the final result.

## Results

### Discriminative brain regions and subgraphs

Betweenness of 90 nodes was calculated from the training sets and two sample *t*-test was performed to evaluate its discriminative power for identifying the EMCI from NC. Betweenness of 90 nodes and *p*-value of two sample *t*-test are shown in Supplemental Text S1. Table 2 lists the 10 discriminative regions (*p* < 0.05) that were selected based on the betweenness. These discriminative regions were found to be consistent with the previous findings.

**Table 2:** The abnormal brain region of local property.

**Brain region**	**Statistical significance (*P*-value)**
L.Middle frontal gyrus	0.037
R.Rolandic operculum	0.004
L.Supplementary motor area	0.048
L.Anterior cingulate and paracingulate gyri	0.043
L.Median cingulate and paracingulate gyri	0.024
L.Posterior cingulate gyrus	0.035
R.Thalamus	0.018
L.Middle temporal gyrus	0.039
R.Middle temporal gyrus	0.007
R.Inferior temporal gyrus	0.020

**Figure 2:**
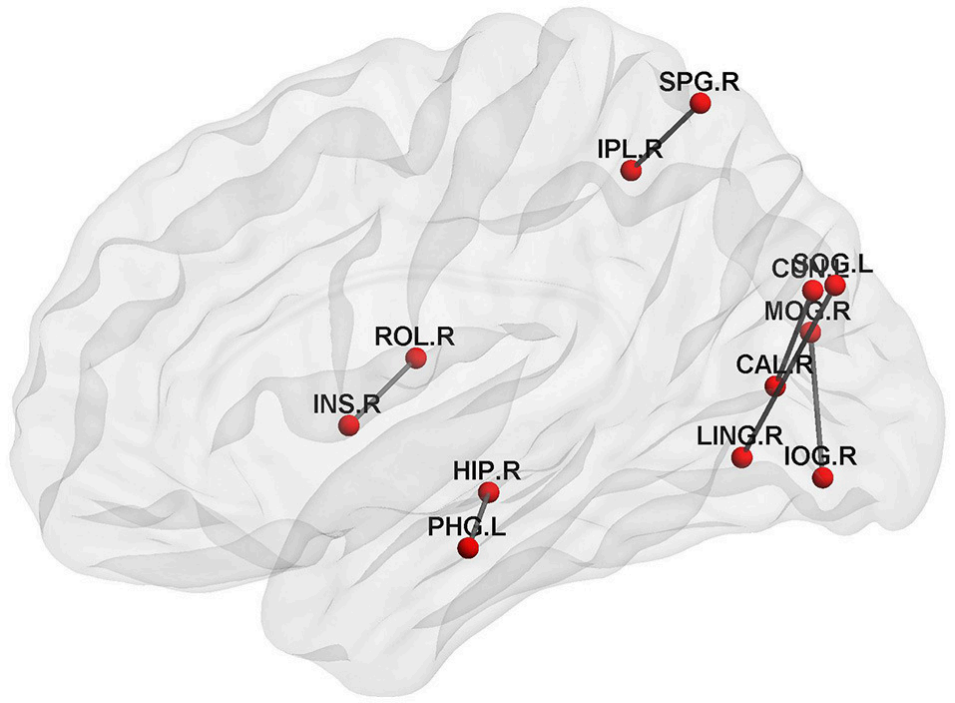
The discriminative subgraphs of EMCI. ROL.R, R Rolandic operculum; INS.R, R Insula; HIP.R, R Hippocampus; PHG.L, L Parahippocampal gyrus; CAL.R, R Calcarine fissure and surrounding cortex; CUN.L, L Cuneus; LING.R, R Lingual gyrus; SOG.L, L Superior occipital gyrus; MOG.R, R Middle occipital gyrus; IOG.R, R Inferior occipital gyrus; SPG.R, R Superior parietal gyrus; IPL.R, R Inferior parietal, but supramarginal and angular gyri.

On the other hand, we also extracted the most discriminative subgraphs based on the global topological property of the training sets. The frequent subgraphs were mined using the gSpan from the MST functional connectivity network of EMCI and NC, with frequency thresholding value of s = 0.7. We obtained 20 frequent subgraphs for EMCI and 22 frequent subgraphs for NC. Then we computed the frequency difference of these subgraphs (Details refer to Supplemental Text S2.), and selected only those subgraphs that exhibited a frequency difference more than 0.13. Thus, we obtained 6 discriminative subgraphs that consisted of 12 abnormal regions. Figure 2 shows the most discriminative subgraphs. Table 3 shows the 12 abnormal brain regions from the subgraph feature.

**Table 3:** The abnormal brain regions of subgraph feature.

**Brain region**
R. Rolandic operculum
R. Insula
R. Calcarine fissure and surrounding cortex
L. Cuneus
R.Middle occipital gyrus
R.Inferior occipital gyrus
R. Superior parietal gyrus
R. Inferior parietal, but supramarginal and angular gyri
R. Lingual gyrus
L. Superior occipital gyrus
R. Hippocampus
L. Parahippocampal gyrus

### Classification performance

In this experiment, the MST was constructed, and the local property and topological property were combined to identify the EMCI from NC. The classification performance was evaluated based on the accuracy, sensitivity, specificity, and area under receiver operating characteristic (ROC) curve (AUC), respectively.

**Figure 3:**
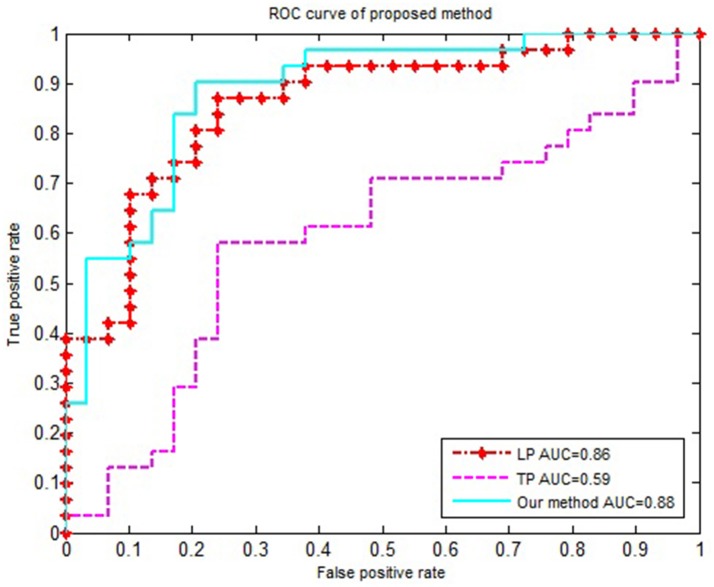
The ROC curve of different methods. The ROC curve of different methods on EMCI vs. NC classification. LP represents the local property is used as classification feature; TP represents the topological property is used as classification feature.

We compared our proposed method using solely the single network property. Specifically, (1) For the local property, denoted as LP, we computed the vector kernel based on the local property. (2) For the topological property, denoted as TP, graph kernel was only computed from the rebuilt networks. All experiments were performed using a 10-fold cross-validation. The classification performances for different methods are summarized in Table 4. Figure 3 shows the ROC curves for these methods.

**Table 4:** The classification performances for different methods.

**Feature**	**ACC (%)**	**SEN (%)**	**SPE (%)**	**AUC**
LP	81.6	86.6	77.5	0.86
TP	61.7	73.3	54.5	0.59
Our method	85	90	79.2	0.88

*ACC, classification accuracy; SEN, sensitivity; SPE, specificity; AUC, the area under the receiver operating characteristic curve; LP represents only use local property as feature to classify; TP represents only use topological property as feature to classify*.

## Discussion

### Discriminative brain regions

Many studies have suggested that the brains of MCI differ from brains of NC in connectivity patterns, such as local properties (Guo et al., 2017), and the topological properties of brain network (Vega-Pons et al., 2014). We used two types of kernels to quantify these two different properties.

On the one hand, the betweenness of the node was calculated to quantize the local property. Then, two sample *t*-test was used to extract the relevant property for classification. From Table 2, we found that (1) compared with NC, the abnormal regions were mainly concentrated in the Default Mode Network (DMN), such as, L. Middle frontal gyrus, L. Anterior cingulate and paracingulate gyri, L. Posterior cingulate gyrus, Middle temporal gyrus, and R. Inferior temporal gyrus. This conclusion is consistent with the view accepted by most researchers that DMN was damaged in the early stages of AD (Garcés et al., 2014; Montembeault et al., 2014; Knh et al., 2017). Similarly, the low-frequency amplitudes of the AD patients were studied and the brain regions were found to be consistent with our study (Yetkin et al., 2006). For example, Liu et al. (2014) found that the low frequency amplitude of AD patients in the bilateral posterior cingulate gyrus, middle temporal gyrus and superior temporal gyrus decreased when compared with the NC. (2) The right Rolandic operculum, the left supplementary motor area and the right thalamus also showed differences, which was consistent with the related literature. Wang et al. (2011) found that the low-frequency amplitude between AD and NC located in the bilateral supplementary motor area and the left fusiform gyrus was different. Yetkin et al. (2006) confirmed that AD was more active in the right middle frontal gyrus, left inferior temporal gyrus, left thalamus, and right lenticular putamen nucleus than the NC. Fei et al. (2014) showed the difference of topological structures between the MCI, and NC were mainly in left rolandic operculum, insula, left supplementary motor area, left hippocampus, left parahippocampal gyrus, right parahippocampal gyrus, and so on.

On the other hand, a graph kernel was calculated to measure the similarity of the topological property. Figure 2 shows that the brain connectivity network changed during EMCI, mainly in the right Rlandic operculum, the right Insula, the right hippocampus, the left parahippocampal gyrus, the right lingual gyrus, the left superior occipital gyrus, the right Calcarine fissure and surrounding cortex, the left cuneus, the right middle occipital gyrus, the right inferior occipital gyrus, the right superior parietal gyrus, and the right inferior parietal. This suggests that the hippocampus, parahippocampal gyrus, and the insula are the first to be damaged in the early stage of AD, which is associated with a decline in memory (Bai et al., 2009), attention, speech, and behavior in early AD patients. Specifically, the hippocampus plays an important role in the spatial memory and in the consolidation of information from short-term memory to long-term memory. The hippocampus demonstrated a significantly negative correlation to episodic memory performance (Bai et al., 2009). The Parahippocampal gyrus plays an important role in the encoding and recognition of environmental scenes (Machulda et al., 2008).

Finally, it can be seen from Table 2 and Figure 2 that the local property and topological property complement each other and provide biomarkers for early diagnosis of MCI from both the local and global aspects.

### Classification performance

A large number of studies have proved that the different features (the local property, the topological property or multi-property) of the traditional threshold network have obtained better classification results. For example, Jie et al. (2014b) constructed multiple threshold connectivity networks of NC and MCI, and extracted the topological features from the multiple threshold connectivity networks. Finally, the multi-kernel SVM was used to classify the two groups of subjects. Fei et al. (2014) had constructed threshold connectivity networks of NC and MCI, and extracted frequent subgraphs, and subsequently selected a discriminative subgraph as a feature. Finally, SVM was used for the classification. These researches show that the subgraph features can better capture the topological information of brain network. Jie et al. (2014a) extracted the local connectivity and global topological properties from five different threshold brain networks and combined these properties by using multikernel SVM for the classification of the MCI and NC. It is shown that the local and topological properties of multi-threshold connected networks were complementary to each other, thus improving the classification performance.

The traditional threshold network construction method is influenced by the threshold, which makes the brain network to exhibit some deviation. In order to avoid these deviations, the MST method was used to construct an unbiased brain network, and this method exhibited a less computational cost, and at the same time, retained the neurological interpretability of the network.

In order to accurately compare the classification performance of different features, we used the same data set, constructed the MST brain network, and calculated the classification performance of the local property, topological property and multi-property feature respectively. Table 4 and Figure 3 showed that our method in combination with the local and topological properties based on MST brain functional network performed significantly better than the single network property. Specifically, for the classification of EMCI and NC, the proposed method achieved 85% accuracy, 90% sensitivity, 79.2% specificity, and 0.88 AUC in the classification. These results show that the proposed classification framework constructed an unbiased brain functional network, captured and combined the local and topological properties, and achieved better classification performance. Compared with the traditional threshold method, our method offered two advantages by avoiding the need to select an optimal threshold, and by making the full use of local and topological properties.

### Effect of the frequency difference threshold T

A large number of frequent subgraphs were obtained by using the gSpan module. In order to select the most discriminative subgraphs, we computed the frequency differences of each of the frequent subgraphs. Then frequent subgraphs with a frequency difference greater than T were considered to be the most discriminative subgraphs. In order to test the effect of the frequency difference threshold (T) on the classification performance, T (the range from 0.06 to 0.13 at a step size of 0.01) was tested separately in the experiment, and the results are shown in the Table 5. The results showed that when T is 0.13, the number of subgraphs was 6, and the classification performance was best. On analyzing six subgraphs, we found that two of them were frequent subgraphs of the EMCI group and the other four were frequent subgraphs of the NC group. Additionally, we also found that the discriminative subgraphs obtained by T = 0. 5 and T = 0. 13 were the same, and they could be the only frequent subgraphs of EMCI or NC. Thus, when T = 0.5, we can obtain the most discriminative subgraphs. In future research, 0.5 can be used as a reference value for T.

**Table 5:** The classification performance and the number of subgraphs under the different parameter T.

**T**	**The number of subgraphs**	**ACC (%)**	**SEN (%)**	**SPE (%)**
0.06	14	83.3	83	83.2
0.07	13	83.3	87.1	80.83
0.08	12	83.3	87.5	77.5
0.09	8	83.3	82.2	79.7
0.1/0.11/0.12	7	81.6	89.3	80.6
0.13	6	85	90	79

*T represents the frequency differences threshold; ACC, classification accuracy; SEN, sensitivity; SPE, specificity*.

### Effect of parameter h

When performing a graph kernel calculation, the number of iterations (h) needs to be set. In the subtree, h represents the height of a subtree. The height of a subtree is the maximum distance between the root and any other node in the subtree. Different h values result in different values of graph kernels. In order to test the effect of parameter h on classification performance, h (*h* ∈ {2, 4, 6, 8, 10}) was tested separately in the experiment, and the results are shown in the Table 6. The results showed that when h = 2 or h = 6, the classification performance was the best. But from the point of view of running time, when h = 2, the running time was shorter.

**Table 6:** The classification performance and the running time under different parameter h.

**h**	**ACC (%)**	**SEN (%)**	**SPE (%)**	**Runtime(s)**
2	85	90	79.2	0.41
4	83.3	84.2	75.5	0.58
6	85	89.1	80	0.76
8	83.3	85.5	70.7	0.97
10	81.7	86.7	77.5	1.11

*h, the number of iteration; ACC, classification accuracy; SEN, sensitivity; SPE, specificity; Runtime, the running time (second) of WL- subtree kernel*.

### Effect of parameter β

**Figure 4:**
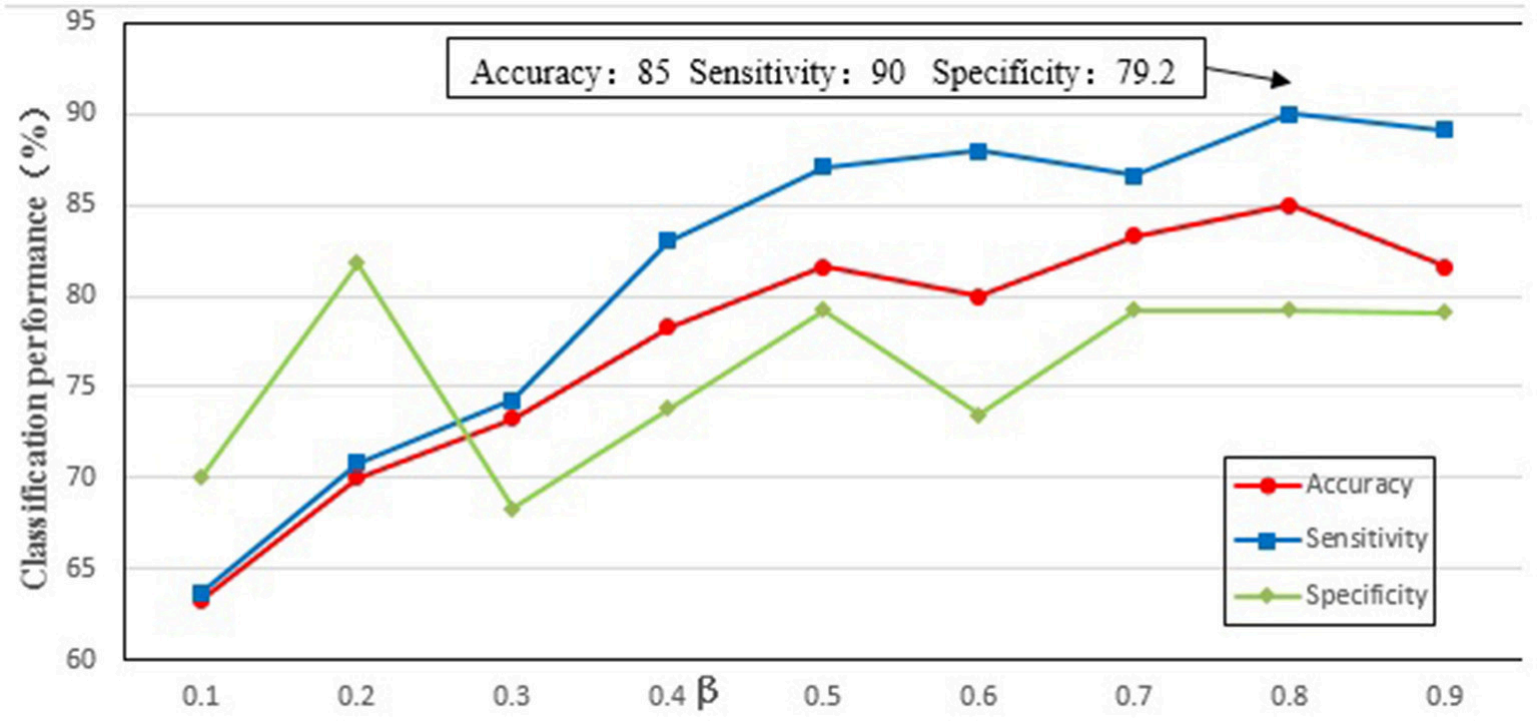
The classification performance of different parameter β. The ordinate indicates accuracy, specificity and sensibility of this method, and the abscissa denotes different parameter β. As shown in the figure, when β = 0.8, better classification performance was obtained, including that accuracy is 85%, and specificity is 90%, and then sensibility is 79.2%.

We needed to find an optimal weighting parameter β in the MKL method. In order to test the effect of weighting parameter β on classification performance, β (the range from 0.1 to 0.9 at a step size of 0.1) was tested separately in the experiment, and the results are shown in Figure 4. It can be seen from Figure 4 that when β is 0.8 the best classification performance is obtained, with 85% accuracy, 90% sensitivity, and 79.2% specificity. The results indicate that the local property (i.e., betweenness) is more important than the topological property of the MST brain functional network for the classification.

### Limitations of the study

This study is limited by the following factors. First, during the network construction, defining of the nodes is a critical step. Previous studies have demonstrated that network nodes can be defined using different brain atlases and image voxels, and the constructed network exhibited different network properties (Hayasaka and Laurienti, 2010). The impact of different brain parcellation atlases on the classification performance will be explored in the future. A second limitation is due to the small amount of data used in the experiment, the results of the classification lack a generality. This method will be applied to larger AD dataset in future work.

## Conclusion

In this paper, we proposed a classification framework based on the MST brain functional connectivity network to identify the EMCI patients and NC. The proposed method mainly used the MST, vector kernel, graph kernel and the multikernel SVM. Specifically, MST was used to construct the brain functional connectivity network; vector kernel was used to extract local property, graph kernel was used to extract global topological property, and the multikernel SVM was adopted to fuse these heterogeneous kernels for classification. In experiments with the ADNI dataset, our proposed method not only significantly improved the classification performance in terms of accuracy, sensitivity, specificity, and AUC value, but also potentially detected the ROIs that are sensitive in the disease pathology.

## Author contributions

XC designed the classification framework and wrote the manuscript. YN performed data analysis and statistical processing. JhX made experiment and gave the proof of results. JX and BW supervised the paper. JC was the head of the funds and supervised the paper. All authors approved the final version of the manuscript.

### Conflict of interest statement

The authors declare that the research was conducted in the absence of any commercial or financial relationships that could be construed as a potential conflict of interest.
