# Supplementary material for: Integrating the Local Property and Topological Structure in the Minimum Spanning Tree Brain Functional Network for Classification of Early Mild Cognitive Impairment
Source: Front Neurosci. 2018 Oct 8;12:701. doi: 10.3389/fnins.2018.00701 (PMC6186843; doi:10.3389/fnins.2018.00701)
Supplement: Supplementary file 1 [file Table_1.DOCX]

Results of two sample t-test for the betweenness of nodes in EMCI and NC. In the table, *p* indicates the level of statistical significance. Significant difference(p<0.05) are indicated in **bold**.

| Labels | Regions | p |
| --- | --- | --- |
| 1 | Precental gyrus | 0.17281 |
| 2 | Precental gyrus | 0.97784 |
| 3 | Superior frontal gyrus, dorsolateral | 0.61504 |
| 4 | Superior frontal gyrus, dorsolateral | 0.67279 |
| 5 | Superior frontal gyrus, orbital part | 0.05256 |
| 6 | Superior frontal gyrus, orbital part | 0.97202 |
| 7 | Middle frontal gyrus | **0.03733** |
| 8 | Middle frontal gyrus | 0.76417 |
| 9 | Middle frontal gyrus, orbital part | 0.96866 |
| 10 | Middle frontal gyrus, orbital part | 0.76449 |
| 11 | Inferior frontal gyrus, opercular part | 0.06890 |
| 12 | Inferior frontal gyrus, opercular part | 0.36968 |
| 13 | Inferior frontal gyrus, triangular part | 0.44464 |
| 14 | Inferior frontal gyrus, triangular part | 0.80862 |
| 15 | Inferior frontal gyrus, orbital part | 0.21543 |
| 16 | Inferior frontal gyrus, orbital part | 0.32787 |
| 17 | Rolandic operculum | 0.35328 |
| 18 | Rolandic operculum | **0.00433** |
| 19 | Supplementary motor area | **0.04818** |
| 20 | Supplementary motor area | 0.24081 |
| 21 | Olfactory cortex | 0.34381 |
| 22 | Olfactory cortex | 0.62259 |
| 23 | Superior frontal gyrus, medial | 0.94337 |
| 24 | Superior frontal gyrus, medial | 0.18678 |
| 25 | Superior frontal gyrus, medial orbital | 0.78069 |
| 26 | Superior frontal gyrus, medial orbital | 0.69020 |
| 27 | Gyrus rectus | 0.98839 |
| 28 | Gyrus rectus | 0.68148 |
| 29 | Insula | 0.82769 |
| 30 | Insula | 0.59073 |
| 31 | Anterior cingulate and paracingulate gyri | **0.04300** |
| 32 | Anterior cingulate and paracingulate gyri | 0.52094 |
| 33 | Median cingulate and paracingulate gyri | **0.02491** |
| 34 | Median cingulate and paracingulate gyri | 0.48029 |
| 35 | Posterior cingulate gyrus | **0.03588** |
| 36 | Posterior cingulate gyrus | 0.05343 |
| 37 | Hippocampus | 0.58055 |
| 38 | Hippocampus | 0.69359 |
| 39 | Parahippocampal gyrus | 0.92035 |
| 40 | Parahippocampal gyrus | 0.73232 |
| 41 | Amygdala | 0.85385 |
| 42 | Amygdala | 0.77637 |
| 43 | Calcarine fissure and surrounding cortex | 0.40177 |
| 44 | Calcarine fissure and surrounding cortex | 0.74787 |
| 45 | Cuneus | 0.36115 |
| 46 | Cuneus | 0.95963 |
| 47 | Lingual gyrus | 0.94450 |
| 48 | Lingual gyrus | 0.20046 |
| 49 | Superior occipital gyrus | 0.97337 |
| 50 | Superior occipital gyrus | 0.55717 |
| 51 | Middle occipital gyrus | 0.63919 |
| 52 | Middle occipital gyrus | 0.48217 |
| 53 | Inferior occipital gyrus | 0.49052 |
| 54 | Inferior occipital gyrus | 0.63376 |
| 55 | Fusiform gyrus | 0.88427 |
| 56 | Fusiform gyrus | 0.91338 |
| 57 | Postcentral gyrus | 0.52091 |
| 58 | Postcentral gyrus | 0.93167 |
| 59 | Superior parietal gyrus | 0.93349 |
| 60 | Superior parietal gyrus | 0.08605 |
| 61 | Inferior parietal, but supramarginal and angular gyri | 0.11172 |
| 62 | Inferior parietal, but supramarginal and angular gyri | 0.18427 |
| 63 | Supramarginal gyrus | 0.98042 |
| 64 | Supramarginal gyrus | 0.16142 |
| 65 | Angular gyrus | 0.78266 |
| 66 | Angular gyrus | 0.58952 |
| 67 | Precuneus | 0.14195 |
| 68 | Precuneus | 0.95750 |
| 69 | Paracentral lobule | 0.16300 |
| 70 | Paracentral lobule | 0.45546 |
| 71 | Caudate nucleus | 0.96047 |
| 72 | Caudate nucleus | 0.67805 |
| 73 | Lenticular nucleus, putamen | 0.50569 |
| 74 | Lenticular nucleus, putamen | 0.23192 |
| 75 | Lenticular nucleus, pallidum | 0.78214 |
| 76 | Lenticular nucleus, pallidum | 0.23851 |
| 77 | Thalamus | 0.44007 |
| 78 | Thalamus | **0.01887** |
| 79 | Heschl gyrus | 0.31971 |
| 80 | Heschl gyrus | 0.24750 |
| 81 | Superior temporal gyrus | 0.46874 |
| 82 | Superior temporal gyrus | 0.64050 |
| 83 | Temporal pole: superior temporal gyrus | 0.56729 |
| 84 | Temporal pole: superior temporal gyrus | 0.82821 |
| 85 | Middle temporal gyrus | **0.03929** |
| 86 | Middle temporal gyrus | **0.00747** |
| 87 | Temporal pole: middle temporal gyrus | 0.49914 |
| 88 | Temporal pole: middle temporal gyrus | 0.74781 |
| 89 | Inferior temporal gyrus | 0.28944 |
| 90 | Inferior temporal gyrus | **0.02002** |
