# Supplementary material for: Integrating the Local Property and Topological Structure in the Minimum Spanning Tree Brain Functional Network for Classification of Early Mild Cognitive Impairment
Source: Front Neurosci. 2018 Oct 8;12:701. doi: 10.3389/fnins.2018.00701 (PMC6186843; doi:10.3389/fnins.2018.00701)
Supplement: Supplementary file 2 [file Table_2.DOCX]

Frequent subgraphs of EMCI and NC When frequency thresholding value s =0.7 and frequency difference of these frequent subgraphs. Discriminative subgraphs (frequency difference >0.13) are indicated in **bold**.

| frequent subgraphs | | frequency of subgraph | | frequency difference |
| --- | --- | --- | --- | --- |
|  |  | nc | emci |  |
| Precentral_L | Temporal_Pole_Mid_L | 0.7586207 | 0.741935 | 0.016685206 |
| Precentral_R | Postcentral_R | 0.9310345 | 0.806452 | 0.12458287 |
| Frontal_Sup_L | Parietal_Sup_L | 0.896551724 | 0.806452 | 0.090100111 |
| Frontal_Sup_R | Frontal_Mid_R | 0.862068966 | 0.774194 | 0.087875417 |
| Frontal_Sup_Orb_L | Frontal_Mid_Orb_L | 0.724137931 | 0.741935 | 0.017797553 |
| Rolandic_Oper_R | Insula_R | 0.75862069 | 0 | **0.75862069** |
| Supp_Motor_Area_R | Olfactory_L | 0.896551724 | 0.870968 | 0.025583982 |
| Olfactory_R | Frontal_Sup_Medial_L | 0.793103448 | 0.83871 | 0.045606229 |
| Frontal_Sup_Medial_R | Frontal_Mid_Orb_L | 0.862068966 | 0.903226 | 0.041156841 |
| Frontal_Mid_Orb_R | Rectus_L | 0.965517241 | 0.935484 | 0.03003337 |
| Rectus_R | Insula_L | 0.862068966 | 0.774194 | 0.087875417 |
| Cingulum_Ant_R | Cingulum_Mid_L | 1 | 0.967742 | 0.032258065 |
| Cingulum_Mid_R | Cingulum_Post_L | 0.965517241 | 0.903226 | 0.062291435 |
| Cingulum_Post_R | Hippocampus_L | 1 | 1 | 0 |
| Calcarine_R | Cuneus_L | 0.724137931 | 0 | **0.724137931** |
| Cuneus_R | Lingual_L | 0.793103448 | 0.870968 | 0.077864294 |
| Occipital_Mid_L | Occipital_Inf_L | 0.862068966 | 0.774194 | 0.087875417 |
| Occipital_Mid_R | Occipital_Inf_R | 0.689655172 | 0 | **0.689655172** |
| Parietal_Sup_R | Parietal_Inf_R | 0.75862069 | 0 | **0.75862069** |
| Precuneus_R | Paracentral_Lobule_L | 0.965517241 | 0.967742 | 0.002224694 |
| Paracentral_Lobule_R | Caudate_L | 0.75862069 | 0.709677 | 0.04894327 |
| Thalamus_R | Heschl_L | 0.862068966 | 0.774194 | 0.087875417 |
| Lingual_R | Occipital_Sup_L | 0 | 0.709677 | **0.709677419** |
| Hippocampus_R | ParaHippocampal_L | 0 | 0.677419 | **0.6774194** |
